# Ultrasonic–Microwave-Assisted Extraction of Flavonoids from Citri Reticulatae Pericarpium and Antioxidant Evaluation of Three Purified Flavonoid Compounds

**DOI:** 10.3390/foods15142531

**Published:** 2026-07-17

**Authors:** Min Qian, Yanxin Li, Hao Dong, Weidong Bai, Wenhong Zhao, Hao Jiang, Xiaoyan Liu, Ziqian Chen

**Affiliations:** Guangdong Provincial Key Laboratory of Lingnan Specialty Food Science and Technology, College of Light Industry and Food Sciences, Zhongkai University of Agriculture and Engineering, Guangzhou 510225, China; qianmin311@163.com (M.Q.); nixnayl@163.com (Y.L.);

**Keywords:** Box–Behnken design, natural antioxidants, hesperidin, nobiletin, tangeretin, Citri Reticulatae Pericarpium

## Abstract

The aim of this study was to optimize ultrasonic–microwave cooperative extraction (UMCE) conditions for flavonoids from Citri Reticulatae Pericarpium (CRP) and to evaluate the antioxidant activities of the purified flavonoid compounds. Using a Box–Behnken design for the experimental study, the influence of each variable on yield was determined, and the optimal conditions for the extraction of these compounds were discovered. The optimal parameters for maximum yield were determined to be 160 W ultrasonic power, 630 W microwave power, 68 °C temperature, 40 min, 57% ethanol and 1:20 ratio of solid to liquid. The primary flavonoids present were hesperidin (13.99 mg/g), nobiletin (4.02 mg/g) and tangeretin (3.80 mg/g). The antioxidant test results showed that hesperidin had greater antioxidant activity than both nobiletin and tangeretin. Based on the results of this study, ultrasonic–microwave cooperative extraction was shown to be an effective approach for enhancing flavonoid recovery from CRP. These findings provide useful insights into the extraction and antioxidant properties of CRP flavonoids and may contribute to the future development of value-added applications of CRP resources.

## 1. Introduction

The dried and mature pericarp of Citri Reticulatae Pericarpium (CRP), known as *Citrus reticulata* Blanco, is predominantly cultivated in several Chinese regions including Guangdong, Fujian, Sichuan, Zhejiang, Chongqing, Hunan, and Jiangxi. Notably, among the various producing regions, Xinhui CRP is widely recognized for its distinctive chemical composition and quality characteristics [1,2]. CRP has been widely used in herbal medicine, flavored beverages, and functional foods owing to its rich composition of bioactive constituents, including flavonoids, volatile oils, alkaloids, limonins, and phenolic acids [3,4,5]. Among these, flavonoids, including hesperidin, nobiletin, tangeretin, and neohesperidin, are the major bioactive components of CRP. Previous studies have demonstrated that CRP flavonoids possess various biological activities, including antioxidant, anti-inflammatory, and anti-asthmatic effects [6,7,8]. Therefore, optimizing the extraction process of flavonoids from CRP is essential to enhance its therapeutic applications and further develop its potential in various industries.

The development of efficient extraction methods is crucial to enhancing the extraction rate and ensuring CRP safety. A variety of techniques, including enzyme-assisted extraction, microwave-assisted extraction, ultrasound-assisted extraction, maceration and Soxhlet extraction, have traditionally been used to extract flavonoids from diverse natural sources [9,10,11]. However, research on the application of ultrasound–microwave cooperative extraction for flavonoid extraction from CRP remains limited. This cooperative method combines ultrasonic cavitation and microwave heating to enhance cell wall disruption, accelerate mass transfer, and facilitate the release of intracellular flavonoids [12,13,14]. Compared to single extraction methods, the ultrasound–microwave cooperative extraction approach requires a more comprehensive consideration of factors and precise power matching. Previous research has shown that inadequate power settings may reduce extraction efficiency, whereas excessive power may lead to degradation of target flavonoids or co-extraction of undesirable compounds [15]. Therefore, optimization of the extraction procedure through systematic experimentation is essential. Several techniques have been developed to achieve more selective enrichment of bioactive compounds in plant extracts, such as solid-phase extraction, liquid–liquid extraction, and high-speed reflux chromatography [16,17,18]. Although these methods can effectively extract primary flavonoid compounds, their capacity and yield remain limited. In contrast, preparative liquid chromatography offers a viable alternative for extracting plant-derived bioactive ingredients owing to its high separation efficiency, large processing capacity, and ability to differentiate substances with similar properties [19,20,21]. Moreover, preparative liquid chromatography has found widespread industrial applications in the isolation and preparation of bioactive extracts rich in flavonoids, glycosides, and saponins [22,23].

Although ultrasound-assisted extraction and microwave-assisted extraction have been widely applied for the recovery of flavonoids from plant materials, studies focusing on ultrasonic–microwave cooperative extraction of flavonoids from CRP remain limited. Furthermore, systematic optimization of extraction parameters combined with purification, structural identification, and antioxidant evaluation of the major flavonoid constituents has not been comprehensively investigated. Unlike previous studies that mainly focused on extraction optimization or the analysis of crude extracts, the present study integrates extraction optimization, purification, structural identification, and antioxidant evaluation of the major flavonoids from CRP within a single workflow. In addition, ultrasonic–microwave cooperative extraction was systematically optimized using response surface methodology and compared with conventional extraction approaches. This integrated strategy provides a more comprehensive understanding of flavonoid recovery and bioactivity from CRP and expands the current research on the utilization of CRP flavonoids. Based on the above considerations, ultrasonic–microwave cooperative extraction was hypothesized to improve flavonoid recovery from CRP, and the purified flavonoid compounds were expected to exhibit distinct antioxidant activities.

Therefore, the objectives of this study were to optimize ultrasonic–microwave cooperative extraction conditions for flavonoids from CRP using response surface methodology, to isolate and identify the major flavonoid compounds by chromatographic and spectroscopic techniques, and to evaluate their in vitro antioxidant activities. The first phase was to use a response surface methodology (RSM), a statistical experimental design, to establish and optimize the dominant parameters of the ultrasonic–microwave extraction technique, which were: ultrasonic power, microwave power, extraction temperature, extraction time, solvent solubility and solid–liquid ratio. Flavonoid compounds were purified from CRP and characterized through LC and also analyzed using nuclear magnetic resonance (NMR) spectroscopy. Following the characterization of the purified flavonoid compounds, their antioxidant activity was evaluated in vitro with respect to 1,1-diphenyl-2-pyridinium free radical, hydroxyl radical scavenging, reducing capacity, and total antioxidant capacity. Ultimately, these studies support the continued exploration and utilization of CRP as an additional source of natural products.

## 2. Materials and Methods

### 2.1. Chemicals and Reagents

The CRP samples used in this study were identified according to the Chinese Pharmacopeia (2020 edition) and further confirmed by experienced practitioners based on appearance, aroma, and labeling information. The samples were obtained from a single farmer in Xinhui District, Jiangmen City, Guangdong Province, China (22.52° N, 113.03° E), the core production area, and originated from the same harvest batch of *Citrus reticulata* ‘Chachiensis’ (harvested in December) to ensure consistency and representativeness. Ten-year-aged CRP was chosen for its recognized status as a representative commercial grade with stable chemical composition and high flavonoid content. After collection, the samples were dried at 60 °C to constant weight (moisture content <13%) and stored in airtight containers at 20–25 °C with relative humidity below 60% until analysis.

Analytical-grade ethanol, methanol, ferric chloride, zinc sulfate, copper sulfate, potassium persulfate, and sodium hydroxide were purchased from Tianjin Damao Chemical Reagent Factory (Tianjin, China). Sodium nitrite (reference grade) was obtained from Tianjin Kermel Chemical Reagent Co., Ltd. (Tianjin, China). Ferric nitrate nonahydrate, dimethyl sulfoxide (DMSO), thiobarbituric acid (>99%), trifluoroacetic acid (TFA), and 2,4-dinitrophenylhydrazine (98%) were purchased from Macklin Biochemical Co., Ltd. (Shanghai, China). Acetonitrile, formic acid, and trifluoroacetic acid (HPLC grade) were obtained from Sigma-Aldrich (St. Louis, MO, USA). DPPH, ABTS, Trolox, hesperidin, nobiletin, and tangeretin standards were purchased from Yuanye Biotechnology Co., Ltd. (Shanghai, China). All reagents used in this study were of analytical grade unless otherwise specified.

### 2.2. Experimental Instruments

The major instruments used in this study included an ultrasonic–microwave cooperative extraction system (XO-SM100, Nanjing Xianou Instrument Manufacturing Co., Ltd., Nanjing, China), a preparative chromatography system (SP1, Shanghai Baitaiqi Trading Co., Ltd., Shanghai, China), an Agilent 1290 Infinity liquid chromatography system coupled with a 6420 Triple Quadrupole mass spectrometer and an Agilent 1260 Infinity HPLC system (Agilent Technologies, Santa Clara, CA, USA).

### 2.3. Sample Preparation

CRP samples were dried in a forced-air drying oven at 60 °C until constant weight was achieved. The dried samples were then ground into powder, passed through a 60-mesh sieve, and stored in airtight containers for subsequent analyses.

### 2.4. Determination of Flavonoid Content

Spectrophotometric analysis combined with a sodium nitrite and aluminum nitrate colorimetric assay, with absorbance measured at 510 nm, enabled the quantification of flavonoids within CRP [24]. A standard curve was established with rutin as the standard reference compound. The linear relationship of absorbance (y) versus rutin standard concentration (C, mg/mL) was represented by a y = 4.76 C + 0.0031 equation. The flavonoid yield was determined using the formula below:

$$\mathrm{Yield}\;\mathrm{of}\;\mathrm{flavonoids}\%=\frac{C\times \mathrm{Extraction}\;\mathrm{volume}\;(\mathrm{mL})\times \mathrm{Dilution}\;\mathrm{ratio}}{\mathrm{Mass}\;\mathrm{of}\;\mathrm{CRP}\;\mathrm{sample}\;(g)}\times 100\%$$

where *C* is the flavonoid concentration (mg/mL), the *extraction volume* is expressed in mL, the *dilution ratio* is dimensionless, and the *mass of the CRP sample* is expressed in g. The flavonoid yield was expressed as percentage (%) relative to the dry weight of the CRP sample.

### 2.5. Ultrasonic–Microwave Cooperative Extraction of Flavonoids from CRP

#### 2.5.1. Experiment of Single Factor

The ranges of ultrasonic power, microwave power, extraction temperature, extraction time, ethanol concentration, and solid–liquid ratio were selected based on previous studies on flavonoid extraction using ultrasonic-assisted, microwave-assisted, and ultrasonic–microwave cooperative extraction techniques. These ranges were chosen to cover commonly reported extraction conditions for plant flavonoids and to allow systematic evaluation of the effects of each factor on flavonoid yield. Preliminary single-factor experiments were conducted to confirm the suitability of these factor ranges and to avoid conditions associated with insufficient extraction efficiency or possible flavonoid degradation.

The effects of ultrasonic power (A), microwave power (B), extraction temperature (C), extraction time (D), ethanol concentration (E), and solid–liquid ratio (F) on the flavonoid yield from CRP were examined. 1.0 g sample of CRP was dissolved in an ethanol solution. Single-factor tests were performed by varying the following parameters: ultrasonic power (80, 120, 160, 200, and 240 W), microwave power (500, 550, 600, 650, and 700 W), extraction temperature (40, 50, 60, 70, and 80 °C), extraction time (10, 20, 30, 40, and 50 min), ethanol concentration (40, 50, 60, 70, and 80%), and solid–liquid ratio (1:10, 1:15, 1:20, 1:25, and 1:30) (Table S1).

#### 2.5.2. Plackett Burman Test Design

Based on single-factor experiments, a Plackett–Burman test was employed to identify the factors that most significantly affected the flavonoid yield from CRP. The factor-level combinations selected for the tests are listed in Table S2.

#### 2.5.3. Response Surface Test Design

Following the results of the Plackett–Burman test, four independent variables (microwave power, extraction temperature, extraction time, and ethanol concentration) were selected to further optimize the flavonoid yield from CRP (Table S3). The Box–Behnken design (BBD) for these four independent parameters yielded 29 experimental runs, including five center points. Each run was performed in triplicate, and the center points were used to estimate experimental error and assess model adequacy.

### 2.6. Isolation, Purification and Structural Identification

#### 2.6.1. Separation and Purification of Flavonoids from CRP

Crude flavonoids extracted from CRP were separated and purified using a fast preparative liquid chromatography system equipped with a RediSep Gold C_18_ column (150 g). The mobile phase consisted of 0.01% trifluoroacetic acid (TFA) in water (solvent A) and acetonitrile (solvent B). Gradient elution was performed as follows: 0–25 min, 10–70% B; 25–30 min, 70–100% B; and 30–35 min, 100% B. The injection volume was 20 μL, and the flow rate was maintained at 30 mL/min. Eluted compounds were monitored at 220 and 240 nm. Fractions containing the major flavonoid compounds were collected according to their chromatographic profiles, combined when corresponding to the same component, concentrated, and used for subsequent analyses.

#### 2.6.2. HPLC Analysis of Flavonoids

Flavonoid composition was analyzed using a Waters XBridge C_18_ column (5 μm, 4.6 mm × 250 mm). The mobile phase consisted of 0.02% TFA in water (solvent A) and 0.02% TFA in acetonitrile (solvent B). The flow rate was set at 1.2 mL/min, the column temperature was maintained at 40 °C, and the injection volume was 2.0 μL. Detection was performed at 283 nm.

The gradient elution program was as follows: 0.0–0.8 min, 90% A and 10% B; 0.8–7.0 min, linear gradient from 90% A to 0% A and from 10% B to 100% B; 7.0–13.0 min, 100% B; and 14.3–20.0 min, 90% A and 10% B. The major flavonoid compounds were identified by comparing their chromatographic retention characteristics, LC–MS molecular mass data, and 1H NMR spectra with those reported in the literature.

#### 2.6.3. Nuclear Magnetic Resonance Analysis

Purified monomers F1, F2, and F3 (2 mg each) were dissolved in deuterated solvent (0.5 mL) and transferred into 5 mm NMR tubes. 1H NMR spectra were recorded on a 400 MHz NMR spectrometer (Bruker AVANCE III, Bruker BioSpin, Rheinstetten, Germany) at room temperature. Structural identification was performed by comparing the obtained spectral data with published literature reports.

### 2.7. In Vitro Antioxidant Assays

#### 2.7.1. DPPH· Free Radical Scavenging Test

The method used to evaluate scavengers of DPPH· is based on the work of Du et al. [25] with some minor modifications. A 0.15 mmoL/L DPPH· standard solution was made with the use of anhydrous ethanol as we have done in the past so as to maintain consistency between standards. To evaluate the DPPH· scavenging activity of the CRP samples, 150 µL of the DPPH· standard solution was mixed with 50 µL (diluted in methanol) of CRP extracts at 5 different concentration ratios (0–1 mg/mL). The mixtures were then stored in dark conditions for 60 min before being evaluated for absorbance at 517 nm. Scavenging activity of DPPH· was then determined using the following formula:

$$\begin{array}{l} \mathrm{DPPH}\;\mathrm{free}\;\mathrm{radical}\;\mathrm{scavenging}\;\mathrm{rate}\;/\;\%=\frac{A_{0}-A_{1}}{A_{0}}\times 100\% \end{array}$$

where A_0_ denotes the absorbance of the methanol control (without the CRP sample) and A_1_ represents the absorbance of the CRP sample.

#### 2.7.2. Abts^+^· Free Radical Scavenging Test

ABTS^+^· inhibition was calculated according to the procedure outlined by Deseo et al. [26] with minor alterations. Mixing equal volumes of both K_2_S_2_O_8_ (at 2.6 mmol/L) and ABTS (at 7.4 mmol/L) produced an incubation solution that was kept at room temperature overnight (12 h). Different grades of CRP extracts were created by using 50 μL of CRP extract and mixing it with 1 mL of the working solution, followed by incubation for 60 min at room temperature. The absorbance of each sample was then measured using an enzyme labeler at a wavelength of 734 nm. Finally, the amount of color produced was measured based on the formula below:

$$\begin{array}{l} \mathrm{ABTS}\;\mathrm{free}\;\mathrm{radical}\;\mathrm{scavenging}\;\mathrm{rate}\;/\;\%=\frac{A_{2}-A_{3}}{A_{2}}\times 100\% \end{array}$$

where A_2_ represents the absorbance of the methanol control (without the CRP sample) and A_3_ is the absorbance of the CRP sample.

#### 2.7.3. Determination of ·OH Radical Scavenging Capacity

A 1.5 mmol/L salicylic acid solution was prepared by dissolving salicylic acid in anhydrous ethanol. The hydroxyl radical (·OH) scavenging ability was determined by adding 2.0 mL of CRP extract, 50 µL of FeSO_4_ (6.0 mmol/L) solution, and 10 µL of H_2_O_2_ (0.1% solution), followed by 0.2 mL of salicylic acid solution in that order, allowing incubation at 37°C for 15 min after which the samples were read at 510 nm for absorbance level. The ·OH scavenging activity was then calculated from the formula:

$$\begin{array}{l} \mathrm{OH}\;\mathrm{free}\;\mathrm{radical}\;\mathrm{clearance}\;/\;\%=\frac{A_{4}-A_{5}}{A_{4}}\times 100\% \end{array}$$

where A_4_ represents the absorbance measured with methanol instead of the CRP sample and A_5_ corresponds to the absorbance of the CRP sample.

#### 2.7.4. Determination of the Reducing Capacity

The reducing capacity of the CRP samples was assessed using the ferric reducing antioxidant power (FRAP) assay following a previously reported method with slight modifications [27]. Briefly, a 0.3 mol/L acetic acid buffer solution (pH 3.6), a 10 mmol/L TPTZ solution, and a 20.0 mmol/L FeCl_3_ solution were prepared. The FRAP working solution was freshly prepared by mixing acetate buffer, TPTZ solution, and FeCl_3_ solution at a volume ratio of 10:1:1. Next, 50 µL of each CRP sample was added to 100 µL of FeSO_4_ solution (6 mmol/L), and 100 µL of salicylic acid solution (6 mmol/L); the mixture was then incubated at room temperature for 10 min. Following incubation, 2 mL of H_2_O_2_ solution (6 mmol/L) was added, and allowed to stand at 30° C for 30 min. Absorbance was measured at 593 nm, while distilled water served as a blank. A standard curve was created from the absorbance (Y) plotted against the molar concentration of FeSO_4_ (X), and a regression line for the standard curve was created and determined to be Y = 0.47744X − 0.289 (R^2^ = 0.999).

### 2.8. Statistical Analysis

All experiments, including extraction optimization, purification, and antioxidant activity assays, were performed in triplicate as independent experimental replicates. Results are expressed as mean ± standard deviation (SD). One-way analysis of variance (ANOVA) was used to evaluate differences among groups, and *p* < 0.05 was considered statistically significant. Statistical analyses and response surface modeling were performed using SPSS Statistics 26.0 (IBM Corp., Armonk, NY, USA) and Design-Expert 13.0 (Stat-Ease Inc., Minneapolis, MN, USA).

## 3. Results and Discussion

### 3.1. Effects of Operation Factors on the Yield of Flavonoids from CRP

#### 3.1.1. Single-Factor Tests

**Figure 1:**
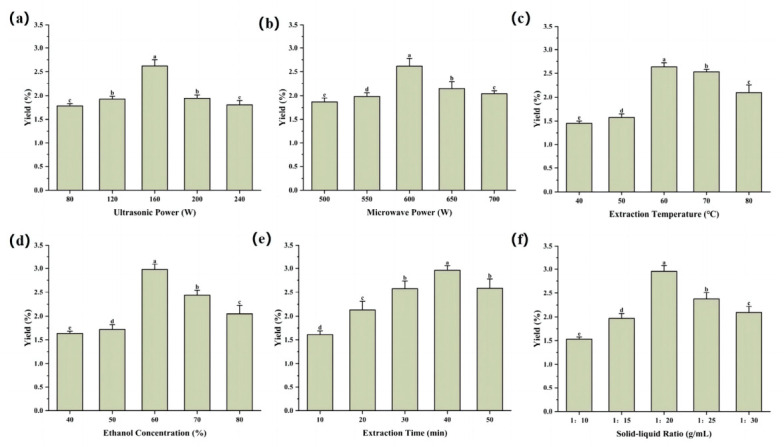
Single-factor test for the extraction of flavonoids from CRP. (**a**) Ultrasonic power; (**b**) Microwave power; (**c**) Extraction temperature; (**d**) Ethanol concentration; (**e**) Extraction time; (**f**) Solid-to-liquid ratio. Different lowercase letters above the bars indicate statistically significant differences (*p* < 0.05).

Figure 1 depicts the effect of different variables (including ultrasonic power, microwave power, extraction temperature, extraction time, ethanol concentration, and solid-liquid ratio) on the yield of total flavonoids in CRP. Increasing the power of the ultrasound machine or the microwave increased the amount of flavonoids extracted. However, when ultrasonic power exceeded 160 W, or microwave power exceeded 600 W, the amount of flavonoids extracted decreased (Figure 1a,b). These results suggest that moderate power levels are beneficial for disrupting the cell-protective layers and facilitating the release of flavonoids. The enhancement of extraction efficiency can be attributed to the synergistic effects of ultrasonic cavitation and microwave dielectric heating. Ultrasonic treatment generates cavitation bubbles that collapse near plant tissues, promoting cell wall disruption and improving solvent penetration, whereas microwave irradiation produces rapid volumetric heating through dipole rotation and ionic conduction, thereby accelerating the release of intracellular flavonoids. In contrast, excessive ultrasonic or microwave power may reduce extraction efficiency due to excessive energy input, which may lead to degradation of flavonoids or reduced mass transfer efficiency.

The extraction temperature, which is a critical factor in active compound isolation, was examined within the range of 40–80 °C while maintaining the other parameters constant. As depicted in Figure 1c, the flavonoid extraction rate increased from 40 °C to 60 °C but subsequently decreased from 70 °C to 80 °C. This pattern indicates that an optimal temperature enhances flavonoid mobility and mass transfer in CRP, thereby facilitating the diffusion of flavonoids into the extraction solvent. However, excessively high temperatures may negatively affect extraction efficiency because prolonged thermal exposure can promote the degradation or structural transformation of heat-sensitive flavonoid compounds. Moreover, the flavonoid extraction rate exhibited a sharp increase during the initial 40 min, followed by a gradual decline to 2.59% at 50 min. This phenomenon may be attributed to the progressive fragmentation of CRP cell-protective structures, which facilitates the precipitation and dissolution of cellular contents in the extraction solution (Figure 1d).

Ethanol concentration and solid–liquid ratio are known to influence flavonoid extraction efficiency. The flavonoid yield initially increased from 1.64% to 2.98% as the ethanol concentration increased from 40% to 60%, and then decreased to 2.05% at 80% ethanol concentration (Figure 1e). Similarly, the solid–liquid ratio exhibited a comparable trend, reaching a peak of 2.96% at a 1:20 ratio (Figure 1f). These findings indicate that moderate ethanol concentrations and solid–liquid ratios are optimal for improving extraction efficiency. This effect is closely related to solvent polarity, which strongly influences flavonoid solubility. Ethanol–water mixtures of intermediate concentration provide an appropriate polarity balance for dissolving flavonoids while maintaining efficient penetration into plant tissues. In contrast, excessively high ethanol concentrations may reduce matrix swelling and hinder mass transfer, resulting in lower extraction yields.

#### 3.1.2. Response Surface Method Was Used to Optimize the Extraction of Flavonoids from CRP

##### Plackett–Burman Test Design Results

Following single-factor experiments, six variables were selected for a series of 12 trials: ultrasonic power (A), microwave power (B), extraction temperature (C), extraction time (D), ethanol concentration (E) and solid–liquid ratio (F). As shown in Table S4, the extraction rate of flavonoids varied between 1.644% and 3.149%. Using Design Expert 13 software, the influence of extraction efficiency for flavonoids by various factors was determined. According to Table 1, ethanol concentration, extraction temperature, extraction time and microwave power are effecting the rate of flavonoid extraction; whereas, solid–liquid ratio and ultrasonic power had virtually no impact on these rates of extraction. ANOVA analysis showed ethanol concentration to be the largest contributor to the rate of extraction, followed by extraction temperature, extraction time and microwave power contribution to extraction. These four factors will be used in future response surface methodology experiments for optimization purposes, while solid–liquid ratio and ultrasonic power will not be examined further because of their negligible impact on flavonoid extraction.

##### The Box–Behnken Test

The effects of the four independent variables (B, microwave power; C, extraction temperature; D, extraction time; and E, ethanol concentration) and their interactions on the flavonoid extraction rate were analyzed using the response surfaces generated by Design Expert 13 after model fitting. As shown in Table S5, flavonoid extraction rates ranged from 1.681% to 3.856%. The quadratic model for the extraction rate, derived from the response surface methodology through multiple regression analyses of the experimental data, is expressed as follows:

Flavonoids Yield = −200.7803 + 0.3938 B + 0.0406 C + 1.0432 D + 2.0325 E + 0.0014 BC –0.0002 BD – 0.0015 BE – 0.0016 CD – 0.0031 CE – 0.0034 DE – 0.0003 B^2^ – 0.0050 C^2^ – 0.0073 D^2^ − 0.0066 E^2^

Variance analysis revealed that the quadratic model was highly significant, whereas the lack-of-fit error term was not significant (Table 2). Factors C (ultramicro temperature), D (extraction time), E (ethanol concentration), and their interactions (BC, BE, CE, DE, B^2^, C^2^, D^2,^ and E^2^) significantly influenced the flavonoid extraction rate (*p* < 0.05).

**Figure 2:**
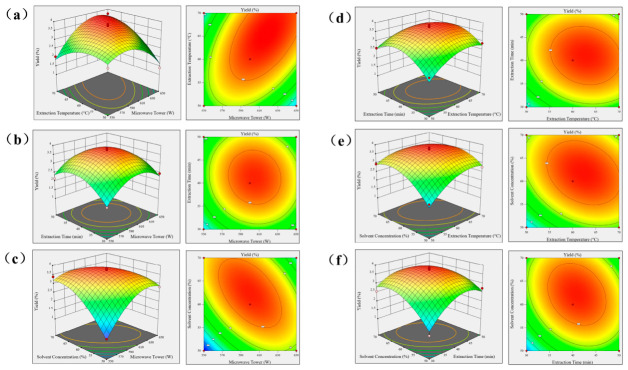
Surface plot and contour plot of the influence of the interaction of various factors on the extraction amount of flavonoids in CRP. (**a**) Microwave power and ultramicrotemperature; (**b**) Microwave power and ultramicroscopic time; (**c**) Microwave power and ethanol concentration; (**d**) ultramicrotemperature and ultramicrotime; (**e**) ultramicrotemperature and ethanol concentration; and (**f**) ultramicroscopic time and ethanol concentration. The color gradient indicates the flavonoid yield (%), with red representing higher values and blue/green representing lower values.

A comprehensive analysis of varying microwave power, ultramicro temperature, ultramicroscopic time and ethanol concentration, as depicted in the four-dimensional representation in Figure 2, revealed their combined effects on flavonoid yield. The ethanol concentration exhibited the most substantial impact, with noticeable changes and large fluctuations in the response surface, indicating that ethanol concentration had the greatest effect on flavonoid yield. This could be attributed to the fact that ethanol concentration directly affects the polarity of the extraction solvent, thereby affecting the solubility and distribution of flavonoids during extraction. Furthermore, response surface analysis highlighted the significant interaction between extraction temperature and extraction time. When other factors were held constant, large fluctuations in the surface and contour line plots indicated that varying the extraction temperature and time affected the dissolution and migration rates of the flavonoids, consequently influencing the yield. In contrast, the microwave power-related sections exhibited relatively smooth fluctuations, suggesting a lower influence of microwave power on the yield of flavonoids, which is consistent with previous results. These results indicate that ethanol concentration, extraction temperature, and extraction time play critical roles in determining flavonoid yield, offering valuable insights for further optimization of the extraction process and yield enhancement.

These findings further demonstrate the importance of ethanol concentration in determining flavonoid extraction efficiency. Ethanol concentration directly influences solvent polarity, which plays a crucial role in the solubility and mass transfer of flavonoids during extraction. In addition, the significant interaction between extraction temperature and extraction time suggests that efficient extraction requires a balance between enhanced mass transfer and prolonged exposure to extraction conditions. Excessively high temperatures or extended extraction times may not further improve flavonoid recovery. Therefore, optimization of these parameters is essential for maximizing extraction yield. These results provide practical guidance for selecting appropriate extraction conditions and support the application of ultrasonic–microwave cooperative extraction as an efficient strategy for flavonoid recovery from CRP.

##### Verification Experiment

To assess the accuracy of the model equation in predicting the optimal response value, a verification experiment was conducted using the derived optimal conditions. As shown in Table S6, the average yield of flavonoids was 3.96% ± 0.18% (*n* = 3) under optimal conditions, slightly exceeding the predicted value of 3.86%. This indicates that the optimized extraction parameters are both accurate and reliable with significant practical implications.

Table S7 shows that the flavonoid yield was 2.33% and 1.85% after ultrasonic and microwave treatments, respectively. However, the extraction rate of flavonoids reached 3.96% under the optimized combined conditions, suggesting that the synergistic effect of ultrasound and microwave treatment was more effective in breaking the cell wall and promoting flavonoid dissolution. These findings highlight the advantages of ultrasonic and microwave-assisted extraction of flavonoids from CRP, offering a promising new approach for improving extraction efficiency.

To further assess the optimized UMCE process, representative extraction methods for citrus peel flavonoids reported in the literature were compared (Table 3). Conventional solvent extraction using 50% ethanol at 40 °C for 20 h typically yields only 0.0092 g/100 g DM of hesperidin [28]. In contrast, single ultrasound-assisted extraction of navel orange peel achieves a total flavonoid yield of 3.16%, but requires a total extraction time of 120 min [29]. Deep eutectic solvent-based UAE methods can provide much higher monomer yields, such as hesperidin 60 ± 2 mg/g and narirutin 21 ± 2 mg/g, but rely on synthesized DES systems that limit scale-up feasibility [30]. Similarly, DIC-Tripolium techniques can improve hesperidin recovery to 1.55–4.67 times that of conventional extraction, but the method requires complex high-pressure decompression equipment [31]. In contrast, the optimized UMCE method in this study achieved a total flavonoid yield of 3.96% within only 40 min, using food-grade ethanol and standard ultrasound–microwave instruments. Overall, these comparisons indicate that the UMCE strategy demonstrates certain advantages in extraction efficiency and processing time, and has good applicability in terms of operational simplicity, showing potential as an effective extraction method. However, its energy consumption, costs, and the feasibility of large-scale application still require further systematic evaluation.

### 3.2. Isolation and Structural Identification of CRP

**Figure 3:**
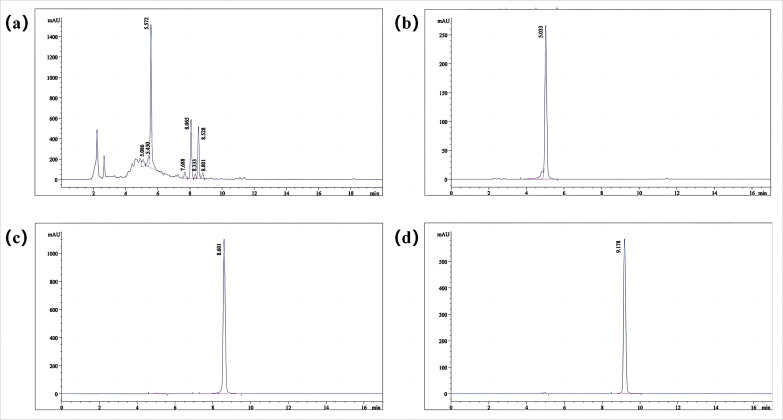
CRP flavonoid liquid chromatography. (**a**) Flavonoids; (**b**) Hesperidin; (**c**) Nobiletin; (**d**) Tangeretin.

CRP flavonoids were extracted by vacuum concentration and freeze-drying the CRP extract solution. Subsequent separation of CRP flavonoids using rapid chromatography yielded three distinct compounds: **F1**, **F2**, and **F3** (Figure 3). The purity of these isolated compounds was assessed by HPLC. Although the overall chromatographic profile of the CRP extract appeared complex, each of the compounds (**F1**, **F2**, and **F3**) exhibited a single peak with purity exceeding 90%.

The identities of the three compounds were further investigated by LC-MS. Compound **F1** showed a molecular ion peak at *m*/*z* 611.1 [M + H]^+^, corresponding to the molecular formula C_28_H_34_O_15_, and was tentatively identified as hesperidin based on database matching. Compound **F2** displayed a molecular ion peak at *m*/*z* 403.0 [M + H]^+^, consistent with the molecular formula C_21_H_22_O_8_, and was putatively identified as nobiletin. Compound **F3** exhibited a molecular ion peak at *m*/*z* 373.0 [M + H]^+^, matching the molecular formula C_20_H_20_O_7_, and was suggested to be tangeretin based on database corroboration.

**Figure 4:**
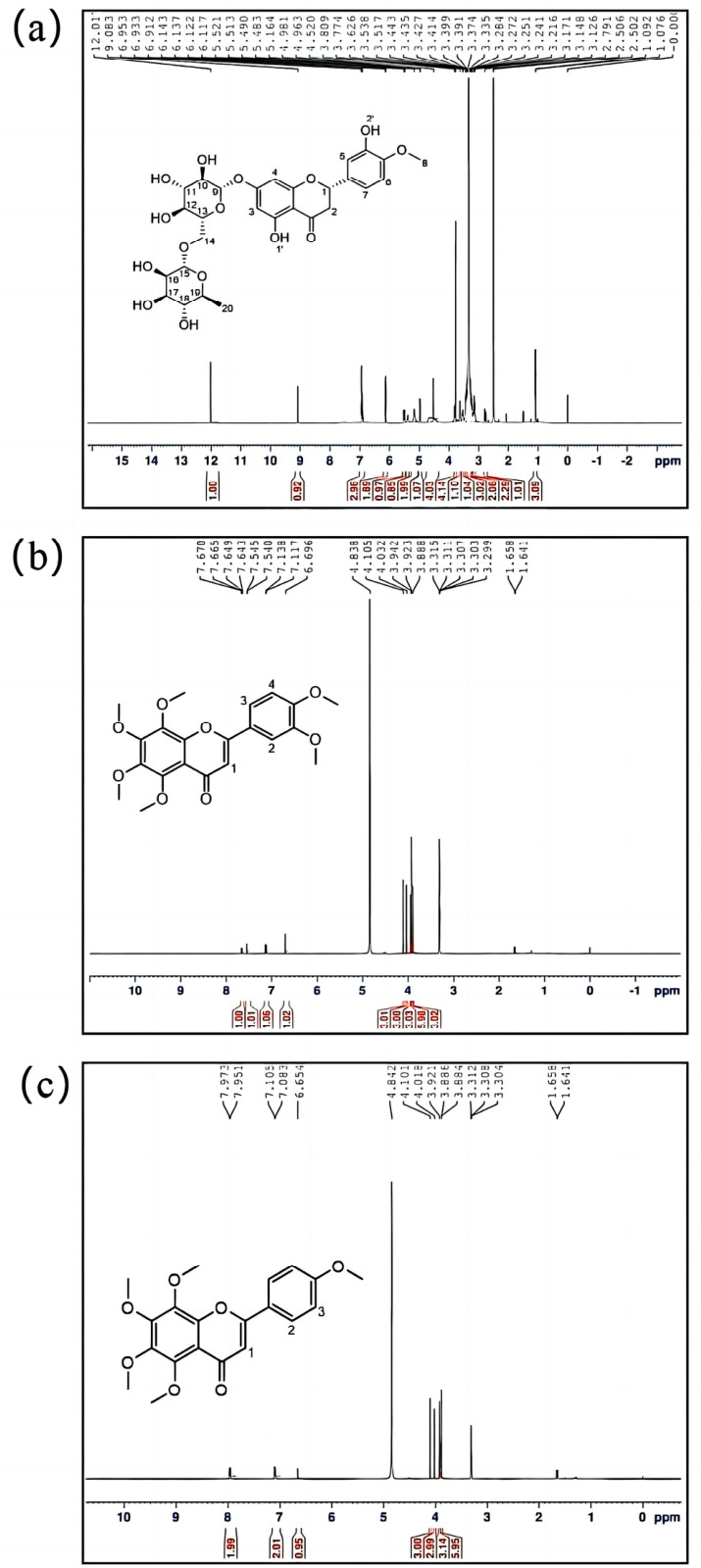
NMR spectra of compound F1 (**a**), F2 (**b**) and F3 (**c**).

Compound **F1**, isolated as a yellow powder, possessed a molecular weight of 610.561 and a molecular formula of C_28_H_34_O_15_. The ^1^H NMR spectrum (400 MHz, DMSO-*d*_6_) of **F1** (Figure 4a) revealed the following signals: δ 12.02 (s, 1H), 9.08 (s, 1H), 6.99–6.89 (m, 3H), 6.18–6.11 (m, 2H), 5.50 (dd, *J* = 12.0, 3.2 Hz, 1H), 5.39 (s, 1H), 5.16 (br s, 2H), 4.97 (d, *J* = 7.2 Hz, 1H), 4.82–4.35 (m, 4H), 3.83–3.76 (m, 4H), 3.66–3.62 (m, 1H), 3.59–3.50 (m, 1H), 3.48–3.42 (m, 3H), 3.41–3.36 (m, 2H), 3.34–3.29 (m, 2H), 2.82–2.75 (m, 1H), 1.08 (d, *J* = 7.2 Hz, 1H). A comparison with literature data confirmed the identity of **F1** as a hesperidin [34].

Compound **F2**, also obtained as a yellow powder, had a molecular weight of 402.39 and a molecular formula of C_21_H_22_O_8_. The ^1^H NMR (400 MHz, MeOD-*d*_4_) of **F2** (Figure 4b) displayed the following signals: δ 7.66 (dd, *J* = 8.4, 2.0 Hz, 1H), 7.54 (d, *J* = 2.0 Hz, 1H), 7.13 (d, *J* = 8.4 Hz, 1H), 6.70 (s, 1H), 4.11 (s, 3H), 4.03 (s, 3H), 3.94 (s, 3H), 3.92 (s, 6H), 3.88 (s, 3H). These spectral data were consistent with literature data, enabling the identification of **F2** as nobiletin [35].

Compound **F3**, isolated as a yellow powder, exhibited a molecular weight of 372.37 and a molecular formula of C_20_H_20_O_7_. The ^1^H NMR (400 MHz, MeOD-*d*_4_) of **F3** (Figure 4c) showed the following signals: δ 7.96 (d, *J* = 8.8 Hz, 2H), 7.09 (d, *J* = 8.8 Hz, 1H), 6.65 (s, 1H), 4.10 (s, 3H), 4.02 (s, 3H), 3.92 (s, 3H), 3.89, 3.88 (s, 6H). These spectral data matched those reported in the literature, confirming that **F3** is tangeretin [36].

The contents of hesperidin, nobiletin, and tangeretin in CRP were determined by HPLC to be 13.99, 4.02, and 3.80 mg/g, respectively. The recoveries of the three compounds ranged from 90.02% to 99.56%. The RSD values for precision, reproducibility, and stability were 0.80–1.14%, 1.01–1.22%, and 0.34–1.70%, respectively (*n* = 6), all below 5%. These results indicate that the established HPLC method is accurate, reproducible, and stable for the quantification of the three flavonoids.

### 3.3. In Vitro Antioxidant Experiments

#### 3.3.1. DPPH· Free Radical Scavenging Activity

**Figure 5:**
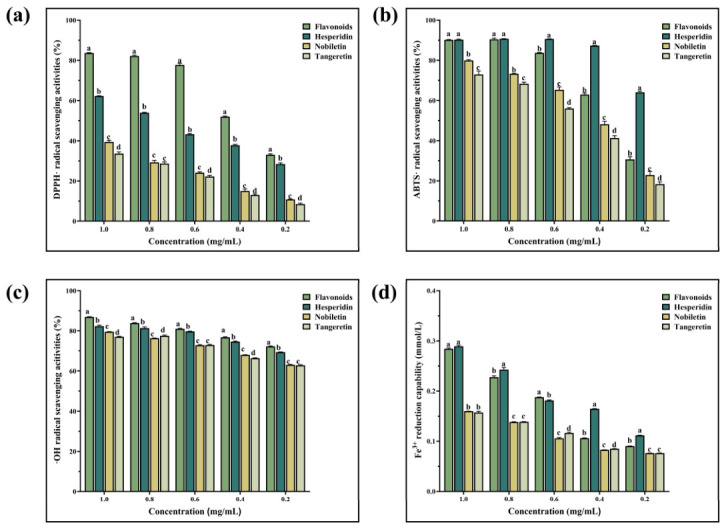
Evaluation of the antioxidant activity of CRP flavonoid extract. (**a**) The ability of CRP flavonoid extract for DPPH· radical clearance; (**b**) Ability of CRP flavonoid extract to clear ABTS^+^· free radicals; (**c**) Ability of CRP flavonoid extract for ·OH radical clearance; (**d**) Fe^3+^ reduction ability of different CRP flavonoid extracts. Different lowercase letters above the bars indicate statistically significant differences (*p* < 0.05).

The DPPH· free radical scavenging test was designed to indirectly assess the antioxidant scavenging capacity of samples by observing color changes that result from the trapping of DPPH· free radicals by trapping agents [37]. The ability of the flavonoids hesperidin, nobiletin, and tangeretin to scavenge DPPH· free radicals at varying concentrations is shown in Figure 5a. At concentrations ranging from 0.2 mg/mL to 1.0 mg/mL, the radical scavenging activity of the flavonoids increased in a dose-dependent manner. Specifically, at a concentration of 1.0 mg/mL, the clearance rates of total flavonoids, hesperidin, nobiletin, and tangeretin were 83.40%, 62.23%, 39.45% and 33.58%, respectively, with corresponding IC_50_ values of 0.29, 0.74, 3.79 and 4.51 mg/mL. These results indicate that both the total flavonoid extract and the purified flavonoid compounds exhibited antioxidant activity. Notably, the scavenging ability of the total flavonoid extract was significantly higher than that of the purified compounds hesperidin, nobiletin, and tangeretin. Moreover, hesperidin demonstrated superior DPPH· free radical-scavenging ability compared to nobiletin and tangeretin. Overall, these findings suggest that both the total flavonoid extract and the purified flavonoid compounds possess substantial antioxidant properties, with hesperidin showing the greatest efficacy in scavenging DPPH· free radicals.

#### 3.3.2. Abts^+^· Free Radical Scavenging Activity

The ABTS^+^· radical scavenging capacities of many flavonoids can be evaluated through the ABTS^+^· radical scavenging assay. Previous studies have evaluated the ABTS^+^· scavenging capacity of several flavonoids [38]. The ABTS^+^· scavenging activities of the flavonoids hesperidin, nobiletin, and tangeretin were assessed (Figure 5b). The results for the ABTS^+^· radical scavenging activities of these flavonoids were comparable to those of the DPPH· scavenging assay, in that the total flavonoid extract and the three purified flavonoid compounds exhibited progressively increasing scavenging capacities as concentrations were increased. Thus, both the total flavonoid extract and the purified flavonoid compounds exhibited significant antioxidant activity. At a concentration of 1.0 mg/mL, hesperidin exhibited 90.19% scavenging ability, while nobiletin and tangeretin exhibited 90.26% and 79.92% and 80.37%, respectively. The IC_50_ values for the total flavonoid extract, hesperidin, nobiletin, and tangeretin that scavenged ABTS+· were 0.26, 0.14, 0.41, and 0.50 mg/mL respectively. When compared to a recent study, the IC_50_ values for Chenpi total flavonoids using the ABTS method range from 0.043 to 0.182 mg/mL [39]. The result (0.26 mg/mL) falls at the upper end of this range, which may be attributable to differences in sample purity. For instance, one study reported that the IC_50_ value of a sample purified by AB-8 macroporous resin was 0.043 mg/mL, significantly lower than the 0.182 mg/mL value of the unpurified sample. Additionally, hesperidin had a significantly higher scavenging effect than nobiletin, tangeretin, and the total flavonoid extract for concentrations between 0.2 and 0.6 mg/mL. However, for the flavonoids and hesperidin, the scavenging effects were similar for concentrations between 0.8 and 1.0 mg/mL.

#### 3.3.3. Determination of ·OH Radical Scavenging Capacity

Hydroxyl radicals (·OH) are extremely reactive and can lead to changes in cells via several mechanisms, including electron transfer and dehydrogenating processes, that result in necrosis, aging, and the proliferation of a tumor [40]. As a result, the ·OH radical scavenging assay is commonly used to measure the antioxidant capacity of many compounds, including some flavonoids such as hesperidin, nobiletin, and tangeretin (Figure 5c). When the concentration of these compounds in CRP was increased from 0.2 mg/mL to 1.0 mg/mL, their scavenging rates for ·OH increased in a dose-dependent manner. At the highest concentration of 1.0 mg/mL, the scavenging rates of total flavonoids, hesperidin, nobiletin, and tangeretin were 86.89%, 82.36%, 79.51% and 77.03%, correspondingly, while their IC_50_ values were 0.014, 0.014, 0.028 and 0.028 mg/mL respectively. Moreover, hesperidin had the greatest ·OH radical-scavenging capacity compared to nobiletin and tangeretin; therefore, these findings suggest that both the total flavonoid extract and the purified flavonoid compounds from CRP have strong hydroxyl radical scavenging activity to scavenge ·OH free radicals with the most noticeable effect being from hesperidin.

#### 3.3.4. FRAP Assay Results

The FRAP assay evaluates antioxidant capacity based on the ability of antioxidants to reduce ferric ions (Fe^3+^) to ferrous ions (Fe^2+^) under acidic conditions. In the presence of 2,4,6-tripyridyl-s-triazine (TPTZ), Fe^2+^ forms a blue-colored Fe^2+^-TPTZ complex, resulting in an increase in absorbance that is proportional to the reducing power of the tested sample. Therefore, the FRAP assay was employed to assess the ferric reducing capacities of the total flavonoid extract and the purified flavonoid compounds derived from CRP [41]. As shown in Figure 5d, the Fe^3+^ reducing abilities of the total flavonoid extract and the purified flavonoid compounds increased gradually with increasing concentration, demonstrating a concentration-dependent relationship. At a concentration of 1.0 mg/mL, the reducing capacities of total flavonoids, hesperidin, nobiletin, and tangeretin were 0.28, 0.29, 0.16 and 0.16 mmol/L, respectively. Notably, hesperidin exhibited stronger Fe^3+^ reducing activity than nobiletin and tangeretin and showed comparable activity to the total flavonoid extract, suggesting its potential role as a potent antioxidant component among the flavonoid constituents in CRP.

Notably, hesperidin exhibited stronger Fe^3+^ reducing activity than nobiletin and tangeretin and showed comparable activity to the total flavonoid extract, suggesting its potential role as a potent antioxidant component among the flavonoid constituents in CRP.

The differences in antioxidant activities among hesperidin, nobiletin, and tangeretin may be attributed to their distinct structural characteristics. Hesperidin is a flavanone glycoside containing multiple phenolic hydroxyl groups capable of donating hydrogen atoms and electrons to neutralize free radicals. These hydroxyl groups facilitate radical stabilization through resonance effects and contribute substantially to antioxidant activity. In contrast, nobiletin and tangeretin are polymethoxylated flavones in which most hydroxyl groups are replaced by methoxy substituents. Although methoxylation may enhance lipophilicity and biological stability, it generally reduces the availability of free hydroxyl groups for direct radical scavenging and electron donation. The replacement of hydroxyl groups by methoxy groups decreases hydrogen-donating capacity and limits direct participation in radical-quenching reactions. Consequently, hesperidin exhibited stronger DPPH radical scavenging activity and ferric reducing power than the polymethoxylated flavones. Interestingly, the differences among the three compounds were less pronounced in the ABTS^+^ and hydroxyl radical scavenging assays, indicating that antioxidant performance depends not only on hydroxyl group content but also on the specific radical system and reaction mechanism involved. In addition, the glycosylated structure of hesperidin may influence its redox behavior and contribute to its antioxidant performance. These findings are consistent with previously reported structure-activity relationships for flavonoids and reflect the combined effects of hydroxylation, methoxylation, glycosylation, and electron-transfer properties.

It should be noted that no standard antioxidant reference compound (e.g., ascorbic acid or Trolox) was included in the present study. Therefore, the antioxidant activities reported here should be interpreted as relative comparisons among the CRP flavonoid extract and the purified flavonoid compounds rather than as absolute measures of antioxidant capacity. Future studies should incorporate standard antioxidant controls to facilitate more comprehensive evaluation and comparison.

## 4. Conclusions

This study employed green extraction techniques, including ultrasonic–microwave collaborative extraction and macroporous resin purification, to enhance the extraction yield of CRP flavonoids. The optimal conditions were established as follows: ultrasonic power of 160 W, microwave power of 630 W, extraction temperature of 68 °C, extraction time of 40 min, ethanol concentration of 57%, and solid–liquid ratio of 1:20. Under these conditions, the extraction yield of CRP flavonoids was 3.96%, which was close to the predicted value of 3.86%. NMR spectroscopy identified hesperidin, nobiletin, and tangeretin as the principal constituents of the CRP flavonoids. Additionally, both the total flavonoid extract and the purified flavonoid compounds effectively mitigated oxidative damage through their antioxidant activities, including DPPH·, ABTS^+^·, and ·OH radical scavenging as well as ferric reducing antioxidant power (FRAP). This study demonstrates the feasibility of the ultrasonic–microwave combined extraction method for extracting total flavonoids from Citri Reticulatae Pericarpium at the laboratory scale. However, certain limitations remain. Since no systematic assessment of the energy consumption, economic cost, or scale-up potential of the method has been conducted, its industrial application prospects require further verification. Subsequent studies will focus on these aspects to comprehensively evaluate the practical application value of this method.

## Figures and Tables

**Table 1:** Evaluation of the factors of the Plackett–Burman trial design.

Project	Quadratic Sum	df	Mean Square	F Value	*p* Value	Significance
model	3.54	6	0.59	18.77	0.0028	
A	0.07	1	0.07	2.09	0.2078	
B	0.37	1	0.37	11.81	0.0185	*
C	1.01	1	1.01	32.23	0.0024	**
D	0.71	1	0.71	22.52	0.0051	**
E	1.22	1	1.22	38.74	0.0016	**
F	0.16	1	0.16	5.23	0.0710	
Residual error	0.16	5	0.03			
Total deviation	3.70	11				

Note: * indicates significant difference (0.01 < *p* < 0.05), ** indicates very significant difference (*p* < 0.01).

**Table 2:** Analysis of variance of the regression equation.

Source of Variation	Quadratic Sum	df	Mean Square	F Value	*p* Value	Significance
model	16.06	14	1.15	25.51	<0.0001	significant
B	0.37	1	0.37	8.26	0.0122	*
C	1.05	1	1.05	23.46	0.0003	**
D	0.49	1	0.49	11.00	0.0051	**
E	1.22	1	1.22	27.11	0.0001	**
BC	1.95	1	1.95	43.46	<0.0001	**
BD	0.05	1	0.05	1.22	0.2885	
BE	2.18	1	2.18	48.51	<0.0001	**
CD	0.10	1	0.10	2.15	0.1646	
CE	0.39	1	0.39	8.60	0.0109	*
DE	0.45	1	0.45	10.09	0.0067	**
B^2^	3.98	1	3.98	88.60	<0.0001	**
C^2^	1.63	1	1.63	36.17	<0.0001	**
D^2^	3.49	1	3.49	77.68	<0.0001	**
E^2^	2.83	1	2.83	63.00	<0.0001	**
residual error	0.63	14	0.05			
Unplanned error	0.57	10	0.06	4.02	0.0962	Not significant
pure error	0.06	4	0.01			
sum	16.69	28				
R^2^	0.96					
R^2^Adj	0.92					

Note: * indicates significant difference (0.01 < *p* < 0.05), ** indicates very significant difference (*p* < 0.01).

**Table 3:** Comparison of flavonoid extraction methods from citrus peels reported in the recent literature.

Extraction Method and Solvent	Key Extraction Conditions	Yield	Notes	References
Conventional solvent extraction (CSE), 50% EtOH	40 °C; 20 h	Hesperidin: 0.0092 g/100 g DM	low yield; long extraction time; used as baseline reference	[28]
Ultrasound-assisted extraction (UAE), 60% EtOH	60 °C; 60 min ultrasonic + 60 min static	Total flavonoids: 3.16%	Moderate yield; long total duration (120 min)	[29]
UAE, 50% EtOH–water	400 W; 30 min	Hesperidin: 113.03 mg/100 g	Green solvent; moderate monomer yield	[32]
Deep eutectic solvent UAE (DES-UAE)	ChCl–lactic acid DES (30% water); 240 W; 28 min	Hesperidin: 60 ± 2 mg/g; Narirutin: 21 ± 2 mg/g	High monomer yield; DES has scale-up limitations	[30]
Microwave-assisted extraction (MAE), 70% EtOH	Up to 140 °C; 8 min	Total flavonoids: 7.6 mg/g; Hesperidin: 58.6 mg/g	High hesperidin recovery; requires MAE reactor.	[33]
DIC–Tripolium eco-extraction	200–600 kPa; 15–45 s cycles	Hesperidin increased 1.55–4.67× vs. CSE	Very efficient; requires high-pressure DIC equipment	[31]
Ultrasound–microwave cooperative extraction (UMCE), EtOH	160 W ultrasound; 630 W microwave; 68 °C; 57% EtOH; 40 min	Total flavonoids: 3.96% ± 0.18%; Hesperidin: 13.99 mg/g	Best balance among yield, extraction time, and equipment accessibility.	This study

## Data Availability

The data presented in this study are available on request from the corresponding authors. The data are not publicly available due to privacy restrictions.

## Supplementary Materials

The following supporting information can be downloaded at: https://www.mdpi.com/article/10.3390/foods15142531/s1, Table S1. Factors and levels in single-factor tests. Table S2. Factors and levels in the Plackett-Burman design. Table S3. Factors and levels in the response surface design. Table S4. Total flavonoid yield from CRP using the Plackett-Burman design. Table S5. Total flavonoid yield from CRP using the response surface design. Table S6. Predicted and actual values of optimal extraction conditions. Table S7. Extraction conditions and corresponding total flavonoid yields from CRP under different pretreatment methods.
