# Supplementary material for: Ultrasonic–Microwave-Assisted Extraction of Flavonoids from Citri Reticulatae Pericarpium and Antioxidant Evaluation of Three Purified Flavonoid Compounds
Source: Foods. 2026 Jul 17;15(14):2531. doi: 10.3390/foods15142531 (PMC13408469; doi:10.3390/foods15142531)
Supplement: Supplementary file 1 [file foods-15-02531-s001.zip › foods-4365482-supplementary.pdf]

Table S1. Factors and levels in single-factor tests.

Level	Factor					
	A (W)	B(W)	C (ºC)	D (min)	E (%)	F (g/mL)
1	80	500	40	10	40	1:10
2	120	550	50	20	50	1:15
3	160	600	60	30	60	1:20
4	200	650	70	40	70	1:25
5	240	700	80	50	80	1:30

Table S2. Factors and levels in the Plackett-Burman design.

Level	Factor					
	A (W)	B (W)	C (ºC)	D (min)	E (%)	F (g/mL)
-1	120	550	50	30	50	1:15
1	200	650	70	50	70	1:25

Table S3. Factors and levels in the response surface design.

Level	Factor			
	B (W)	C (ºC)	D (min)	E (%)
-1	550	50	20	50
0	600	60	30	60
1	650	70	40	70

Table S4. Total flavonoid yield from CRP using the Plackett-Burman design.

No.	A (W)	B (W)	C (ºC)	D (min)	E (%)	F (g/mL)	Yield (%)
1	200	550	70	50	50	1:25	2.974±0.145
2	120	650	70	50	50	1:15	2.228±0.126
3	120	550	70	30	70	1:25	2.905±0.194
4	200	550	50	30	70	1:15	2.167±0.125
5	120	550	50	50	50	1:25	2.330±0.191
6	120	650	70	30	70	1:25	2.914±0.169
7	200	650	50	50	70	1:25	2.353±0.084
8	200	550	70	50	70	1:15	3.149±0.022
9	200	650	70	30	50	1:15	1.687±0.018
10	120	650	50	50	70	1:15	2.537±0.144
11	120	550	50	30	50	1:15	1.644±0.117
12	200	650	50	30	50	1:25	1.340±0.144

Values are expressed as mean ± SD (n = 3). Statistical significance was evaluated by one-way ANOVA at $p < 0.05$.

Table S5. Total flavonoid yield from CRP using the response surface design.

No.	Factor				
	B (W)	C (ºC)	D (min)	E (%)	Yield (%)
1	550	60	40	50	1.112±0.104
2	600	60	30	70	2.552±0.106
3	600	70	30	60	2.865±0.005
4	600	70	40	50	2.762±0.175
5	600	60	40	60	3.701±0.319
6	600	60	40	60	3.805±0.06
7	550	50	40	60	2.479±0.094
8	650	60	40	50	2.811±0.144
9	600	50	50	60	2.583±0.116
10	600	70	40	70	2.647±0.083
11	650	50	40	60	1.382±0.186
12	600	60	40	60	3.545±0.18
13	600	60	50	70	2.656±0.19
14	600	50	30	60	2.065±0.155
15	600	60	40	60	3.752±0.178
16	600	50	40	50	1.806±0.153
17	600	60	40	60	3.856±0.093
18	600	60	30	50	1.267±0.182
19	600	60	50	50	2.718±0.199
20	650	70	40	60	3.736±0.091
21	550	70	40	60	2.037±0.185
22	650	60	30	60	2.448±0.143
23	550	60	40	70	3.383±0.175
24	600	50	40	70	2.935±0.122
25	650	60	50	60	2.448±0.151
26	600	70	50	60	2.761±0.104
27	550	60	30	60	1.681±0.134
28	550	60	50	60	2.149±0.193
29	650	60	40	70	2.128±0.122

Values are expressed as mean ± SD (n = 3). Statistical significance was evaluated by one-way ANOVA at $p < 0.05$.

Table S6. Predicted and actual values of optimal extraction conditions.

	B (W)	C (ºC)	D (min)	E (%)	Yield (%)
predicted value	630.85	68.12	40.76	56.86	3.86
actual value	630.00	68.00	40.00	57.00	3.96±0.18

Values are expressed as mean ± SD (n = 3). Statistical significance was evaluated by one-way ANOVA at $p < 0.05$.

Table S7. Extraction conditions and corresponding total flavonoid yields from CRP under different pretreatment methods.

No.	A (W)	B (W)	C (ºC)	D (min)	E (%)	F (g/mL)	Yield (%)
1	160	630	68	40	57	1:20	3.97±0.15
2	160	0	68	40	57	1:20	2.33±0.16
3	0	630	68	40	57	1:20	1.85±0.14

Values are expressed as mean ± SD (n = 3). Statistical significance was evaluated by one-way ANOVA at $p < 0.05$.
